# The Constitutively Active V2 Receptor Mutants Conferring NSIAD Are Weakly Sensitive to Agonist and Antagonist Regulation

**DOI:** 10.1371/journal.pone.0008383

**Published:** 2009-12-21

**Authors:** Julie Tenenbaum, Mohammed A. Ayoub, Sanja Perkovska, Anne-Laure Adra-Delenne, Christiane Mendre, Bruno Ranchin, Giamperro Bricca, Ghislaine Geelen, Bernard Mouillac, Thierry Durroux, Denis Morin

**Affiliations:** 1 CNRS, UMR 5203, Institut de Génomique Fonctionnelle, Montpellier, France; 2 INSERM, U661, Montpellier, France; 3 Université Montpellier 1,2, Montpellier, France; 4 Centre de Référence des Maladies Rénales Rares, Département de Néphrologie, Rhumatologie, Neurologie HDJ et Dialyse, Hôpital Femme-Mère-Enfant (HFME), Hospices Civils de Lyon, UFR de Médecine Lyon-Est and Inserm U499, Université Claude Bernard Lyon I, Lyon, France; 5 Laboratoire de Pharmacologie, UFR de Médecine Lyon-Est and Inserm ERI 22, Domaine Rockefeller, Université Claude Bernard Lyon I, Lyon, France; 6 Exploration Fonctionnelle Endocrinienne et Métabolique (EFEM), CBN, Hôpital de la Croix Rousse, Hospices Civils de Lyon, Laboratoire de Physiologie, UFR de Médecine Lyon-Est, Lyon, France; 7 Centre de Référence des Maladies Rénales Rares du Sud-Ouest, Néphrologie Pédiatrique CHU Montpellier, Montpellier, France; Institut Europeen de Chimie et Biologie, France

## Abstract

Patients having the nephrogenic syndrome of inappropriate antidiuresis present either the R137C or R137L V2 mutated receptor. While the clinical features have been characterized, the molecular mechanisms of functioning of these two mutants remain elusive. In the present study, we compare the pharmacological properties of R137C and R137L mutants with the wild-type and the V2 D136A receptor, the latter being reported as a highly constitutively active receptor. We have performed binding studies, second messenger measurements and BRET experiments in order to evaluate the affinities of the ligands, their agonist and antagonist properties and the ability of the receptors to recruit β-arrestins, respectively. The R137C and R137L receptors exhibit small constitutive activities regarding the G_s_ protein activation. In addition, these two mutants induce a constitutive β-arrestin recruitment. Of interest, they also exhibit weak sensitivities to agonist and to inverse agonist in term of G_s_ protein coupling and β-arrestin recruitment. The small constitutive activities of the mutants and the weak regulation of their functioning by agonist suggest a poor ability of the antidiuretic function to be adapted to the external stimuli, giving to the environmental factors an importance which can explain some of the phenotypic variability in patients having NSIAD.

## Introduction

Various pathologies have been described as the consequence of G protein-coupled receptor (GPCR) mutations [1]. X-linked congenital nephrogenic diabetes insipidus (cNDI) has thus been described as a loss of function of the vasopressin (AVP) V2 receptor, and more than 200 mutations of the receptor gene have been identified [2]. By contrast to this well characterized pathology, only few patients with nephrogenic syndrome of inappropriate antidiuresis (NSIAD) have been reported [3]–[8]. These patients exhibit hyponatremia, and inappropriate elevated urinary osmolality often associated to low plasma vasopressin levels. Though the clinical and biological features have been described through 9 case reports, much less is known regarding the pharmacological properties of the mutated receptors. In the original study, two mutations, R137C and R137L, responsible for NSIAD and localized in the highly conserved DRY/C motif in GPCR class A have been reported to confer to the receptor a G protein constitutive activity [3].

Here we observed that the G protein constitutive activity is associated to a constitutive recruitment of arrestins, the recruitment of β-arrestins being involved in receptor-G protein desensitization as well as in promoting the activation of G protein-independent signaling pathways [9]–[11]. These two signalling pathways are weakly regulated by AVP and inverse agonist. As such these two mutants differ from the D136A mutant, a constitutive V2 receptor identified only in heterologous expression systems and which exhibit strong constitutive and regulated activities [12].

## Results

**Figure 1:**
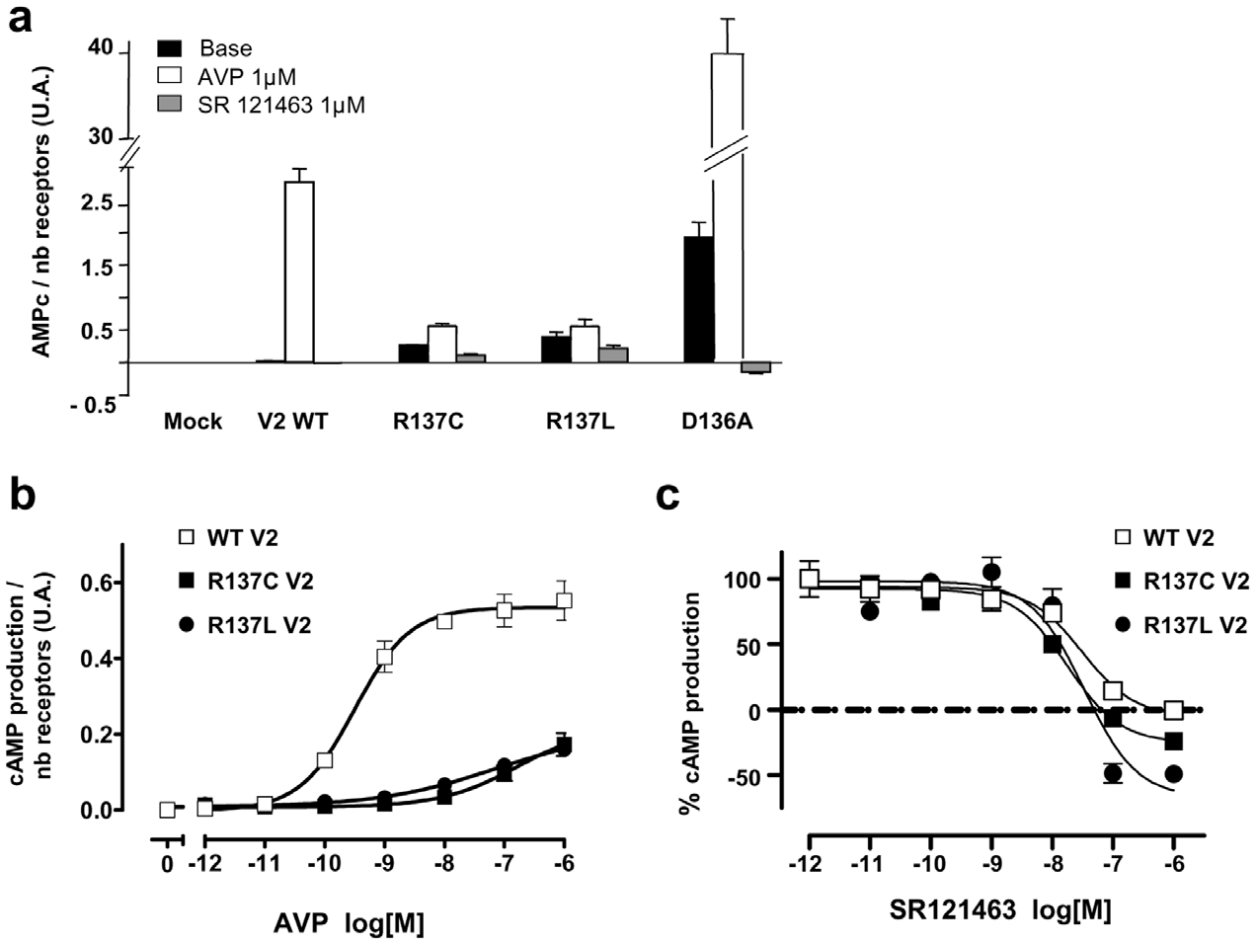
Coupling properties of the wild-type and mutants receptors. **a**, Basal, agonist induced and antagonist-inhibited cAMP accumulation was measured on cos 7 cells expressing wild-type or mutants receptors. Values of cAMP accumulation were normalized to the number of receptors expressed at the surface of the cells determined by ligand binding [3H]AVP. **b**, AVP dose-response experiments performed on cells expressing wild-type, R137C or R137L V2 receptor. **c**, effect of an inverse agonist, SR121463, on AVP-induced stimulation.

The R137C and R137L mutants when expressed in COS-7 cells exhibited a significant constitutive activity when measuring the intracellular cAMP accumulation. The basal cAMP production normalized to the number of cell surface receptors determined by ligand binding was significantly higher in cells expressing R137C or R137L receptors compared to cells expressing the WT receptor (Figure 1a), confirming the constitutive activity measured by the indirect gene reporter assay [3]. These constitutive activities remained of small amplitude compared to that measured with the D136A mutant [12] (Figure 1a). Surprisingly, SR121463, an inverse agonist, hardly decreased the constitutive activities of R137C and R137L receptor compared to the D136A receptor. This absence of large effect was not due to a loss of affinity since competition experiments of [^3^H]AVP binding showed that SR121463 exhibits similar inhibition constant (K_i_) for the wild-type and the mutant receptors (Table 1).

**Table 1:** Pharmacological properties of the R137C and R137L V2 receptors compared to the those of the wild-type and D136A receptor.

	Affinity of AVP	Affinity of SR121463	AVP-induced cAMP production	AVP-induced BRET increase between V2-Rluc and b-arrestin 1-YFP		
Receptor	Kd (nM)	Ki (nM)	EC50 (nM)	BRETmax	T1/2 (min)	EC50 (nM)
Wild-type	2.61±0.44	1.2±0.11*	0.40±0.09	99±5	8.1±1.8	3.3±1.3
R137C	2.8±0.18	1.34±0.32	>100*	47±2	16.1±4.7	46.6±19.5
R137L	3.71±0.17	2.41±0.86	>30*	53±3	17.4±5.6	43.3±15.6
D136A	0.40±0.27	0.79±0.18	0.5±0.15 **	10±2	N.D.	N.D.

* : the values cannot be determined since the curves do not reach a plateau.

** : values from [19].

Then, we have examined the agonist-dependent receptor activity in cAMP assay. Similarly, R137C and R137L receptors presented a weak sensitivity to AVP stimulation, compared to the wild-type receptor, dose-response curves with R137C and R137L mutants exhibiting a very significant rightward shift of AVP potency and a lower efficiency (Figure 1b and Table 1). The differences between WT and mutants were not due to lower affinities of AVP for R137C and R137L mutants as verified by saturation experiments with [^3^H]AVP (Table 1). Of note, the AVP-induced cAMP production was inhibited by increasing concentrations of SR121463 (Figure 1c). Taken together, our data indicate that R137C and R137L mutations did not change the binding properties of V2 receptors but they largely affect the agonist-promoted G protein activation. As such, it differs from the super activity of D136A receptor [12].

**Figure 2:**
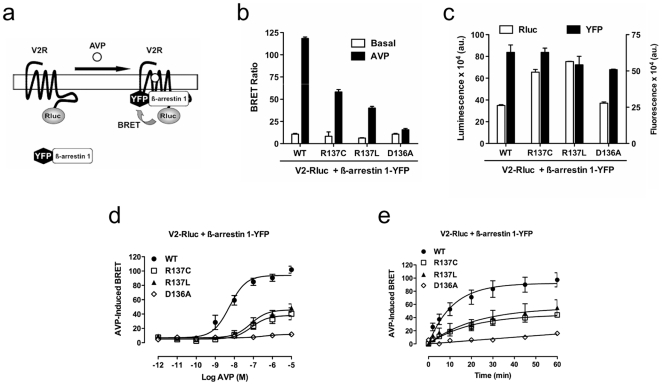
β-arrestin 1 recruitment to V2R studied by BRET. **a**, For BRET measurements, V2 receptors and β-arrestin 1 were fused to Rluc and YFP proteins, respectively, and co-expressed in COS-7 cells treated or not with AVP for 45 minutes. **b**, AVP-induced β-arrestin 1 recruitment to either V2 wild-type, R137C, R137L or D136A mutants. Data are means±S.E.M of three independent experiments. **c**, Expression levels of BRET partners determined by Rluc luminescence and YFP fluorescence **d**, Dose-response of AVP-induced BRET after AVP stimulation for 45 minutes. **e**, BRET time-course: BRET increase between V2 receptors and β-arrestin 1 after AVP stimulation (1 µM) at the indicated time. Data are means±S.E.M of three independent experiments.

Next, we examined the physical association of V2 receptors with β-arrestins, the recruitment of β-arrestins being involved in V2 receptor desensitization [13]. For this, bioluminescence resonance energy transfer (BRET) experiments [9], [14], [15] were performed using V2 chimeric receptors and β-arrestins respectively fused to the luciferase from *Renilla reniformis* (Rluc) and the yellow fluorescent protein (YFP) (Figure 2a). In this configuration, we did not observe any significant basal BRET signal (Figure 2b). By contrast, AVP stimulation induced a significant BRET signal indicating β-arrestin 1 recruitment. Of note the recruitment observed with R137C and R137L receptors is about 50% less than with the wild-type receptor (Figure 2b) despite a slightly higher expression determined by luminescence measurements (figure 2c). Dose-response experiments of AVP-induced BRET showed right-shifted curves with R137C and R137L mutants compared to the wild-type receptor (Figure 2d and Table 1). This observation is consistent with dose-response results in cAMP assay. Moreover, kinetics analysis of AVP-induced BRET increase indicates that t_1/2_ values of β-arrestin 1 recruitment with V2 mutants are twice greater than that obtained with the wild-type receptor (Figure 2e and Table 1). It is noteworthy that the absence of a significant BRET signal with the D136A receptor is most probably due to a very low expression level of the receptor at the cell surface as previously shown [12]. Moreover similar results were obtained with β-arrestin 2 and in HEK293 cell line indicating an absence of β-arrestin or cell line specificity (data not shown).

**Figure 3:**
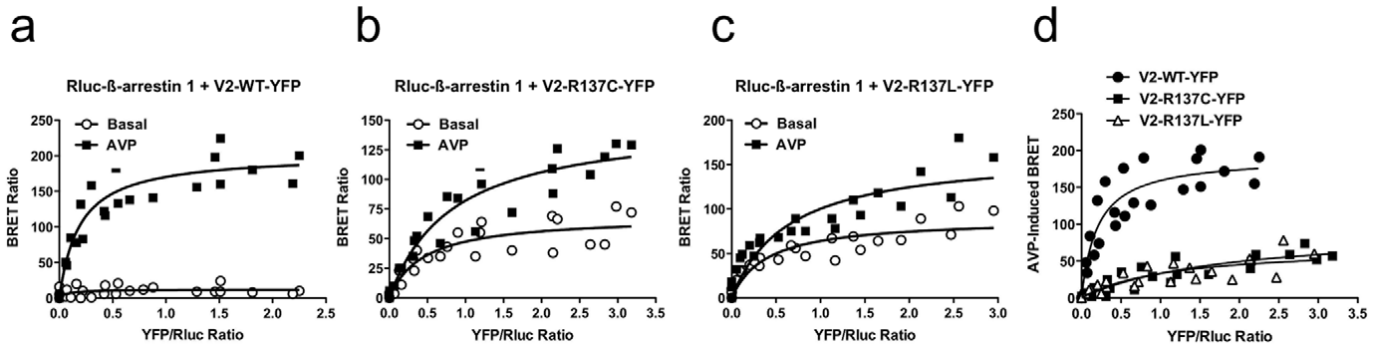
BRET-saturation of basal and AVP-induced β-arrestin 1 recruitment. **a**, The inverse configuration of BRET was used for saturation experiments where V2 receptors and β-arrestin 1 were fused to YFP and Rluc proteins, respectively. BRET measurements were performed in COS-7 cells co-expressing a constant amount of Rluc-β-arrestin 1 with the increasing level of V2-YFP receptors: the wildtype (**a**), R137C (**b**) or R137L (**c**) in the absence (○) or presence (▪) of AVP stimulation (1 µM) for 45 minutes. **d**, AVP-induced BRET increase compared between V2R wildtype, R137C and R137L mutants. BRET_50_ are 0.2, 1.84 and 1.12 for the wild-type, R137C and R137L receptors, respectively. Each panel corresponds to three independent experiments.

Given the constitutive production of cAMP with the mutant receptors, and because V2 receptors have been shown to undergo β-arrestin dependent internalization after their activation {Oakley, 1999 #621}, we were expecting a constitutive recruitment of β-arrestins. Our results were also at variance with those previously reported [16], the discrepancy between the two studies being possibly linked to the level of mutant receptors trapped into the cells, leading to a variation in the luminescent signal in the basal conditions. We then performed BRET experiments with the reverse configuration, *i.e.* V2-YFP receptor and Rluc-β-arrestin 1 (Figure 3). Interestingly, this configuration led us to measure a significant constitutive BRET signal between β-arrestin 1 and R137C or R137L mutants Figure 3b and c), but not with the wild-type V2 receptor (Figure 3a), indicating a basal recruitment of β-arrestin 1 by V2 mutants which could be associated with their constitutive activity. In addition, in this configuration, the BRET saturation experiments indicated that AVP-promoted β-arrestin 1 recruitment reveals first, a lower AVP induced-recruitment of β-arrestin 1 by the mutant receptors than by the wild-type receptor, and second, a lower affinity of β-arrestin 1 for the V2 receptor mutant after AVP stimulation (Figure 3d). The BRET_50_ for the different curves are 0.20, 1.85, 1.12 for the WT, R137C and R137L, respectively. Taken together, these data indicate that, by contrast to the wild-type receptor, R137C and R137L mutants exhibit a constitutive β-arrestin recruitment but they also exhibit a lower sensitivity to AVP stimulation in β-arrestin recruitment.

## Discussion

NSIAD is a rare disease recently described. Two features are particularly interesting, regarding the molecular and the physiological aspects. We observe for the R137C and R137L receptors a correlation between the production of cAMP and β-arrestins recruitment as well as in the basal or AVP-induced stimulation conditions. Both receptors exhibit a rightward shift of the AVP-induced dose-response curves of cAMP production and β-arrestins recruitment. These data are consistent with those reporting that the recruitment of β-arrestin is consecutive to V2 receptor activation [13]. Although R137C and R137L V2 mutants exhibit G protein constitutive activities as the D136A receptor, they displayed different properties. By contrast to the D136A receptor which exhibits a large constitutive activity sensitive to inverse agonist and a super activity when stimulated with agonists [12], the R137C and R137L mutants show small amplitude constitutive activities which are also weakly sensitive to the inverse agonist. This observation is consistent with patient's insensitivity to the administration of inverse agonist [4]. These mutants are thus in an almost blocked conformation. By contrast the small AVP-induced stimulation of receptor is completely inhibited by inverse agonist suggesting that the receptors can also adopt a second more flexible conformation. The existence of two conformations may be responsible for the discrepancies observed in the BRET signal with the two BRET configurations used since energy transfer is dependent on the distance or the orientation of the donor and the acceptor.

NSIAD exhibits a large phenotypic variability by contrast to the cNDI pathology characterized by a clear phenotype. Indeed if few patients have been identified at a early stage [3], [8], others were at a later one [6]. This variability is not reliable to the nature of the mutation since only two mutations, R137C in eight cases and R137L in one case, have been identified. These two mutants exhibit weak constitutive activities suggesting that it can be compatible with life as soon as water balance is maintained in a narrow window. In such conditions, the detection of the pathology is thus difficult. However, environmental factors (heatwave, sports…) can be at the origin of behavioural modifications inducing water intake and then water overload, leading to more severe symptoms. Moreover AVP is detectable in some patients with NSIAD [8] while it is not in others. The presence or the absence of AVP should not have important consequences on the phenotype since the R137C and R137L receptors exhibit only a weak AVP-induced activity

Finally the two mutations responsible for NSIAD contrast with the high number of mutations in cDNI. It suggests that mutations such as D136A which exhibit *in vitro* very high constitutive activity and therefore which should confer more severe symptoms have never been reported since they are probably not compatible with life. However one can speculate that other mutations should be identified. Indeed the DRY/H motif in the third transmembrane domain is engaged with residues of the sixth transmembrane domain in an ionic lock which prevents receptor activation [17]. Therefore mutations in the sixth transmembrane domain can result in the expression of the NSIAD syndrome.

In conclusion, the expression of the R137C and R137L mutants result in a background production of cAMP leading to a constitutive water reabsoption. Since the activity of the receptor is hardly regulated by vasopressin or by inverse agonist, it suggests that these receptors are in a blocked conformation preventing any good adaptation of the antidiuretic activity to environmental stimulation. Therefore it suggests the importance of environmental parameters in the expression of the disease. Although the regulation of the water balance is compatible with life in normal conditions in patient having NSIAD, the repeat of situations leading to water overloading can severely affect patient health. The importance of the environmental parameters can then be responsible in part for the phenotypic variability of NSIAD. The identification of NSIAD syndrome is recent with a small hindsight, but the establishment of precise criteria for early diagnosis of the disease is therefore necessary.

## Materials and Methods

### Cell Culture

Cos-7 cell lines were maintained in culture in Dulbecco's modified Eagle's medium supplemented with 10% fetal calf serum and 100 units/mL penicillin and streptomycin in an atmosphere of 95% air and 5% CO2 at 37°C. Cells were transiently transfected by electroporation with various concentration of vector coding for human V1a receptor and empty vector to a final amount of 10 *í*g. Under this condition, the expression level is in the range of 0.5 to 2 pmol/mg protein. Cells were also transfected with Lipofectamine 2000 (Invitrogen) according to the recommandations of the manufacturer.

### Membrane Preparations

Culture dishes of Cos-7 expressing the human vasopressin V2 receptors were washed twice in PBS without calcium and magnesium, and cold lysis buffer (15 mM Tris:HCl, 2 mM MgCl2, 0.3 mM EDTA, pH 7.4) was added. Cells were scraped with a rubber, homogenized, and centrifuged at 100 *g* for 5 min at 4°C. Supernatants were recovered and centrifuged at 44 000 *g* for 30 min at 4°C. Pellets were resuspended in a suspension medium (50 mM Tris:HCl, 5 mM MgCl2, pH 7.4) centrifuged at 44 000 *g* for 30 min at 4°C. Pellets were resuspended in an appropriate volume of the same buffer. For each membrane preparation, protein content was evaluated, and membranes were then aliquoted and frozen in liquid nitrogen.

### Binding Experiments on Membrane

Binding assays were performed at 30°C using [^3^H]AVP as the radioligand and 5–15 µg of membrane proteins per assay. Affinities of [^3^H]AVP for the various mutants were determined in saturation experiments using concentrations ranging from 0.2 to 15 nM. Affinities for other ligands were determined fro competition experiments using 2–5 nM [^3^H]AVP as radioligand. The concentration of the unlabeled ligands varied from 1 pM to 1 µM. Non specific binding was determined in the presence of vasopressin (1 µM). Bound tritiated vasopressin ([^3^H]AVP) fractions were separated from the free tritiated vasopressin by filtration. We used Whatman GF-C filters preincubated in bovine serum albumin (10 mg/mL). Filtration was performed on a Brandel apparatus. Radioactivity on the filters was counted on a beta-counter Tri-carb 2100TR (Perkin-Elmer Life and Analytical Sciences). The ligand binding data were analyzed by nonlinear least-squares regression using the computer program Graphpad Prism (GraphPad Software). All assays were performed in triplicate on at least three separate batches of electroporated cells.

### Binding Experiments on Cells

Experiments were performed in a 96 well plate format. Cells which were rinsed twice with a cold isotonic saline buffer (NaCl, 146 mM; KCl, 0.42 mM; MgCl_2_, 0.5 mM, CaCl_2_, 1 mM; Hepes, 2 mM), have been incubated for 4 hours at 4°C in the presence of 10 nM [^3^H]AVP, 1 mM phenylalanine and in the presence or the absence of an excess of unlabeled AVP (1 µM). Cells were then rinsed three times with cold isotonic saline buffer and 100 µl of NaOH (0.1N) was added in each well. The lysates were transferred in radioactivity counting vial. NaOH (0.1 N) was used to rinse each well and transfer to the counting vial. 200 µl of HCl (0.1 N) were added in counting vial to neutralized NaOH and radioactivity was counted on beta-counter Tri-carb 2100TR (Perkin-Elmer Life and Analytical Sciences).

#### cAMP measurements

Experiments were performed as previously described Briefly activation/inhibition of cAMP pathways by AVP receptor agonists or antagonists, was determined using cAMP dynamic kits (Cisbio Bioassays, Bagnols-sur-Cèze, France) according to the recommendations of the manufacturer. Cells were incubated in the presence of a phosphodiesterase inhibitor, Ro-20-1724 (Calbiochem, Darmstadt, Germany) to prevent cAMP degradation. The basal cAMP productions were measured after a 5 h-long accumulation period. Agonist activations or antagonist inhibitions were performed as previously described [18]. Dose dependent curves were fitted with Graphpad Prism software. The experiments were repeated at least three times on different cultures. Normalization was performed as indicated in the figure legends, either as a percentage of the maximal value or as a percentage of the maximal value for AVP when comparisons were necessary.

#### BRET measurements

For BRET experiments, V2 receptors and β-arrestin 1 were fused to Rluc and YFP proteins and co-expressed in COS-7 cells. Adherent cells were cultured in 96-well plates and 48 hours after transfection cells were first stimulated with AVP in DMEM-serum free supplemented by BSA 0.1% and then washed with twice PBS. BRET measurements were performed in PBS in 50 µl final volume after addition of coelenterazine h (5 µM) using the Mithras LB940, as previously reported [15].
